# Evaluation of large language models for discovery of gene set function

**Published:** 2023-09-07

**Authors:** Mengzhou Hu, Sahar Alkhairy, Ingoo Lee, Rudolf T. Pillich, Robin Bachelder, Trey Ideker, Dexter Pratt

**Affiliations:** 1 Department of Medicine, University of California San Diego, La Jolla, California, USA; 2 Department of Computer Science and Engineering, University of California San Diego, La Jolla, California, USA

## Abstract

Gene set analysis is a mainstay of functional genomics, but it relies on manually curated databases of gene functions that are incomplete and unaware of biological context. Here we evaluate the ability of OpenAI’s GPT-4, a Large Language Model (LLM), to develop hypotheses about common gene functions from its embedded biomedical knowledge. We created a GPT-4 pipeline to label gene sets with names that summarize their consensus functions, substantiated by analysis text and citations. Benchmarking against named gene sets in the Gene Ontology, GPT-4 generated very similar names in 50% of cases, while in most remaining cases it recovered the name of a more general concept. In gene sets discovered in ‘omics data, GPT-4 names were more informative than gene set enrichment, with supporting statements and citations that largely verified in human review. The ability to rapidly synthesize common gene functions positions LLMs as valuable functional genomics assistants.

## Introduction

A fundamental goal of the ‘omic sciences is to identify the sets of genes that are responsible for all of the distinct biological functions of life, health and disease. In this vein, numerous mRNA expression experiments over the past two decades have produced (and continue to produce) sets of genes that are differentially expressed across conditions or that cluster by expression similarity. Similarly, proteomics experiments produce lists of proteins that are co-abundant, co-modified, or physically interacting; CRISPR gene knockout screens produce lists of genes required for fitness or a particular response, and so on. In all of these cases, the basic premise is that the identified genes work coherently towards the same biological process or function.

To understand these functions, one turns to functional enrichment analysis^1–9^. This approach seeks to identify similarities between the gene sets of interest and those from a separate, pre-defined gene function database, such as the Gene Ontology^10,11^ (GO), Molecular Signature Database^12^ (MSigDB), Kyoto Encyclopedia of Genes and Genomes^13–15^ (KEGG), or Reactome^16–18^, which annotate genes to known functional and pathway categories^17,19–23^.

Paradoxically, a gene set for which there is a very strong enrichment in a gene function database may be of lesser interest, since the set and its function have already been well characterized by previous studies. Of greater interest are gene sets that fail functional enrichment or overlap known functions only marginally, because it is precisely from these ‘failures’ that new biological findings emerge. In these cases, an immediate next step is to explore the biological literature, as well as complementary data sets, to learn as much as possible about the genes in question. The goal is to mine knowledge pertinent to each gene and then use this knowledge to synthesize mechanistic hypotheses for a function that might be held in common by all or most genes in the set. As a result, new functional categories might be defined and added to existing collections of functions. This protracted process of discerning relevant findings from data and literature, then reasoning on this information to synthesize functional hypotheses, has not yet been widely automated but is one of the central tasks performed by a genome scientist.

Highly relevant to these tasks is the recent advent of generative artificial intelligence (AI) models and specifically Large Language Models (LLMs) such as GPT-4^24^ and PaLM^25^, which are reshaping many domains including computational biology. At its core, generative AI is an approach to machine learning in which a model is trained to recognize underlying patterns in data in a manner that allows it to generate new data with similar properties as the training data. The underlying technology behind LLMs is the transformer architecture^26–28^, which uses a self-attention mechanism to understand context and handle long-range dependencies in text, delivering significant advancements in tasks such as text translation, summarization, and generation. Large-scale training of general-purpose LLMs such as GPT-4 incorporates information from an enormous corpus of sources, including the biomedical literature.

Here, we evaluate the degree to which LLMs provide insightful functional analyses of gene sets. First, we develop a gene set analysis pipeline based on queries to GPT-4, as of this writing one of the most capable general LLMs available^24^. We then test the ability of this system to propose succinct names and analysis paragraphs for gene sets of interest. In addition, we formulate a separate GPT-4-based method to orchestrate relevant literature search and to verify statements made in the primary analysis. Finally, we discuss our findings and their implications for the general use of LLMs in functional genomics applications.

## Results

### Development of a GPT-4 functional genomics pipeline.

**Extended Data Fig. 1.**
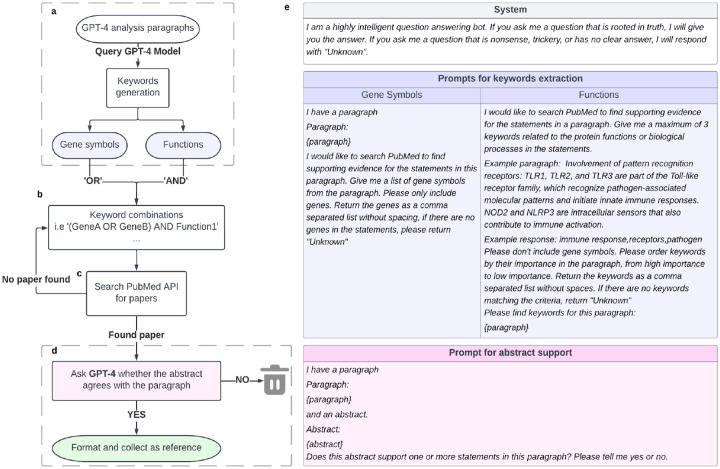
Schematic of the citation module. **a**, GPT-4 is asked to provide gene symbol keywords and functional keywords separately. **b**, Multiple gene keywords are combined with ‘OR’ and then with one function using ‘AND’. **c**, PubMed is searched for relevant titles and/or abstracts in the scientific literature. **d**, GPT-4 is queried to evaluate the abstract, saving supporting references. **e**, Prompts used for each query of the GPT-4 model.

**Fig. 1.**
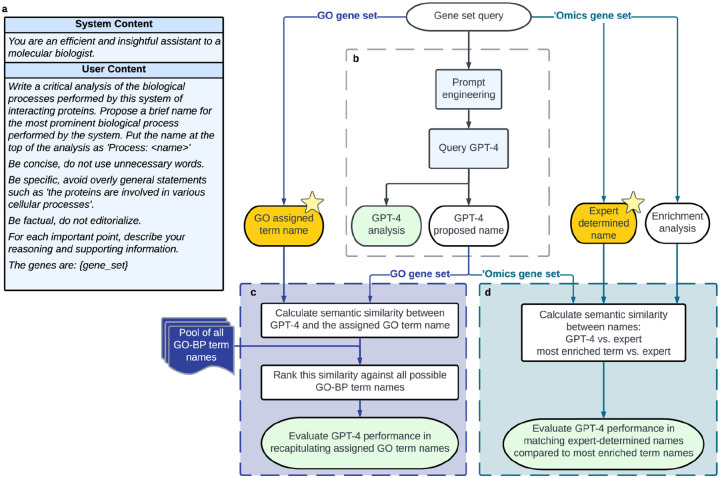
Use and evaluation of GPT-4 for functional analysis of gene sets. **a**, The prompt provided to GPT-4 for its input fields “System” and “User.” The specific list of genes is inserted into the {gene_set} field at the end of the prompt template. **b**, Generation of a proposed name for a gene set, with an accompanying analysis, using GPT-4. **c**, Benchmarking against GO gene sets: Calculation of semantic similarity between the GPT-4 proposed name and the name assigned by the GO curators (Left yellow star). Calibration of this similarity against the distribution of semantic similarities between the GPT-4 name and every term name in GO:BP. **d**, Benchmarking against ‘omics gene sets (MSigDB hallmark gene sets, NeST cancer systems): Computation of semantic similarity between the GPT-4 proposed name and the name curated by human experts (Right yellow star). Additional comparison to the name assigned by gene set enrichment analysis using the Enrichr web service.

We designed a pipeline in which the GPT-4 model is instructed to formulate biologically descriptive names and supporting analysis text for a gene set (Fig. 1a–b). A separate GPT-4-based system was implemented to validate statements made in the analysis text with pertinent literature citations (Extended Data Fig. 1, Methods). The instruction to an LLM is called a “prompt” and can include data and examples to guide the response. Best practices for formulating this prompt are the subject of ongoing experimentation^29–31^, but in this study our prompt simply described desired properties of the name and analysis to be generated, including guiding phrases such as “For each important point, describe your reasoning and supporting information” (Fig. 1a, Extended Data Fig. 1e).

To assess this gene set analysis pipeline, we implemented evaluation workflows that score the quality of names given by GPT-4 and assess statements in the summary analysis text. Reference gene sets were derived from two primary sources. The first was literature curation, for which we evaluated sets of genes drawn from Gene Ontology terms^10,11^ (Workflow 1; Fig. 1c). The second source was ‘omics analysis, for which we evaluated gene sets identified by (1) similarity in mRNA expression data^12^, and (2) modular community detection in protein interaction networks^32^ (Workflow 2; Fig. 1d). In both the literature-curation and ‘omics-analysis workflows, GPT-4 performance for a gene set was measured by the semantic similarity of its proposed name to the name proposed by human scientists. This performance measure, “semantic similarity,” uses a numerical score in the range [0–1] to approximately capture how similar two phrases are to each other in conceptual meaning within a language^33^ (see Online Methods). For example, the word “socks” is semantically closer to the word “shoes” than it is to “airplane.”

### Workflow 1: Benchmarking GPT-4 names against names assigned by GO.

**Extended Data Fig. 2.**
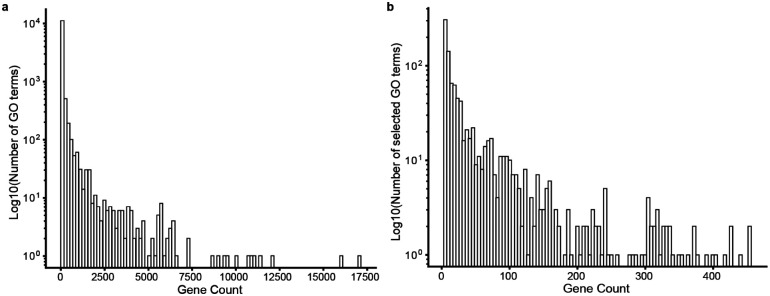
Distribution of GO term gene sizes. **a**, Distribution of number of genes per GO term among all terms in GO:BP (n = 12,214; y axis). **b**, Distribution of number of genes per GO term among selected 1000 GO terms (y axis).

For the first workflow, we queried GPT-4 to propose names and supporting analysis text for 1000 human gene sets drawn randomly from terms in the Gene Ontology Biological Process branch (GO:BP, 2022-10-07 release, sampling terms from 3 to 500 genes; see Extended Data Fig. 2). On average, it took about 70 seconds for the GPT-4 model to process each gene set (Extended Data Table 1), after which time it returned a proposed concise name and supporting analysis text. For example, the gene set annotated to GO term “Acute-Phase Response” (GO:0006953) resulted in GPT-4 proposing a nearly exact match, “Acute Phase Response” (differing from the GO name only in omission of the hyphen). GPT-4 also generated eight paragraphs of supporting text describing the various lines of evidence for specific genes and their common functional roles (Table 1).

**Fig. 2.**
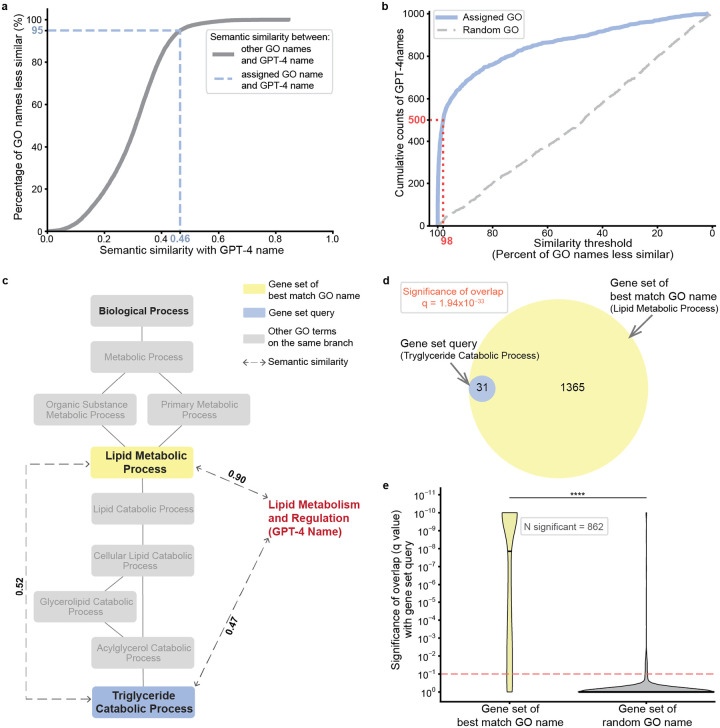
Evaluation of GPT-4 in recovery of GO term names. **a**, The raw semantic similarity between the GPT-4 name and the assigned GO name (blue dashed line, x-axis) is converted to the percentage of all names in the GO database with lower similarity to the GPT-4 name (blue dashed line, y-axis). Plot shown is for the GPT-4 name “DNA Repair and Chromosome Segregation”, generated for the GO term “Regulation of Sister Chromatid Cohesion.” **b**, Cumulative number of GO term names recovered by GPT-4 (y-axis) at a given similarity percentile (x-axis). 0 = least similar, 100 = most similar. Blue curve: semantic similarities between GPT-4 names and assigned GO term names. Grey dashed curve: semantic similarities between GPT-4 names and random GO term names. The red dotted line marks that half of the 1000 sampled GO names are recovered by GPT-4 at a similarity percentile of 98%. **c**, Hierarchical view of the GO term “Triglyceride Catabolic Process” and its ancestors. Blue box: gene set query, yellow box: gene set of best match GO name (most similar GO name to GPT-4 name), dashed lines with arrows: semantic similarities between names, red text: GPT-4 proposed name. **d**, Venn diagram showing overlap between the gene set query (blue, LEFT) and gene set of best match GO name (yellow, RIGHT). The false discovery rate (q-value) is obtained from the hypergeometric test between the two gene sets. **e**, Significance (q-value by hypergeometric test) of overlap between the gene set with the best match GO name and the gene set query (y-axis). Red dashed line: significance cutoff at q = 0.1 (Benjamini-Hochberg correction). Black horizontal line within violin plot shows median q-value (Best match GO: 1.40×10^−8^, Random GO: 1.0). **** p = 1.18×10^−279^ by Mann–Whitney U test.

To quantify the accuracy of name generation, we then computed the semantic similarity between the GPT-4 proposed name and the name assigned by GO. These scores ranged from values as high as 1, in cases where the GPT-4 name was an exact match to the GO name (e.g., “Mitochondrial tRNA Modification”), to values below 0.2, in cases where the names were not intuitively similar (e.g., GPT-4: “Hedgehog Signaling Pathway” versus GO: “Positive Regulation of Protein Import Into Nucleus”) (Table 2 and **Supplementary Table 1**). To calibrate this similarity, we ranked it against a background semantic similarity distribution, defined by comparing the GPT-4 name against all 12,214 term names documented in GO:BP (Fig. 1c, see Online Methods). For example, the GPT-4 name (“DNA Repair and Chromosome Segregation”) had a semantic similarity to the assigned GO name (“Regulation of Sister Chromatid Cohesion”) of 0.46, a score that was higher than 95% of alignments between the GPT-4 name and every other term name in GO:BP (Fig. 2a). Using this scoring approach, we found that exactly half of the gene set names proposed by GPT-4 were close matches to the corresponding GO term names, with semantic similarities ranking in the 98^th^ percentile (500/1000; Fig. 2b).

In many other cases, we observed that GPT-4 had suggested a name matching a more general ancestor in the GO term hierarchy. For example, the gene set annotated to the GO term “Triglyceride Catabolic Process” resulted in the GPT-4 name “Lipid Metabolism and Regulation” with a semantic similarity of 0.47 ranking in the 96^th^ percentile. The GPT-4 name matched most closely to the GO term “Lipid Metabolic Process,” a less specific category higher in the ontology (Fig. 2c) and annotated by a larger set of genes (Fig. 2d). This observation was reinforced by a systematic analysis of the 500 queries for which GPT-4 and GO names were less similar (bottom half by semantic similarity). In 47.2% of these cases, the best matching GO name (i.e., that with the highest semantic similarity to the GPT-4 name) corresponded to a gene set representing a broader ancestral concept in the GO hierarchy. Moreover, across all 1000 queries, the GO terms with the best matching names to GPT-4 often had significant gene set intersection with the actual gene set queries (862/1000 cases or 86.2%, q-value < 0.1, hypergeometric test coupled with Benjamini-Hochberg correction; Fig. 2e). Thus, even when the GPT-4 name is semantically dissimilar to the actual name assigned by GO, it often matches another GO name annotated by a similar, sometimes broader, set of genes.

### Workflow 2: Benchmarking GPT-4 names for ‘omics gene sets.

**Extended Data Fig. 3.**
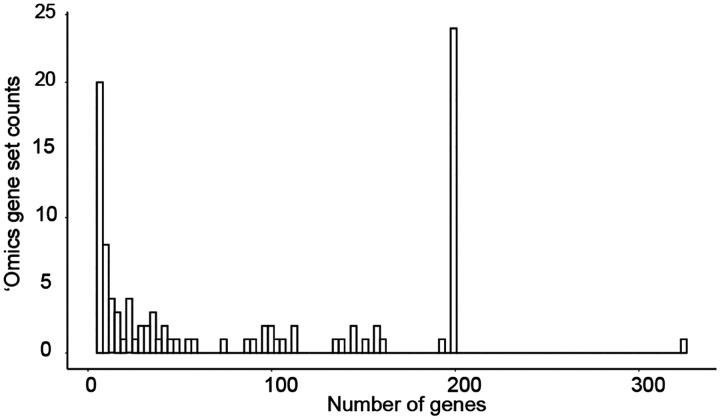
Distribution of ‘omics gene set sizes. Number of genes per gene set among all ‘omics gene sets considered in this study. (n=100).

In workflow 2, we tested the performance of GPT-4 in naming and analyzing 100 gene sets informed by ‘omics approaches. The first 50 of these gene sets, drawn from MSigDB^12^, integrated information from clusters of coordinately expressed genes identified in a broad collection of mRNA transcriptomic studies. Each gene set had been reviewed and named by a panel of human authors based on their prior knowledge of biological processes. The second set of 50 gene sets was drawn from the NeST collection of cancer protein complexes, based on identifying communities of interacting proteins in a large protein interaction network^32^. Here also, the corresponding gene sets had been named by a panel of human experts. Together, these resources included gene sets covering a range of sizes (5 to 323 genes per set; Extended Data Fig. 3 and **Supplementary Table 2**).

**Fig. 3.**
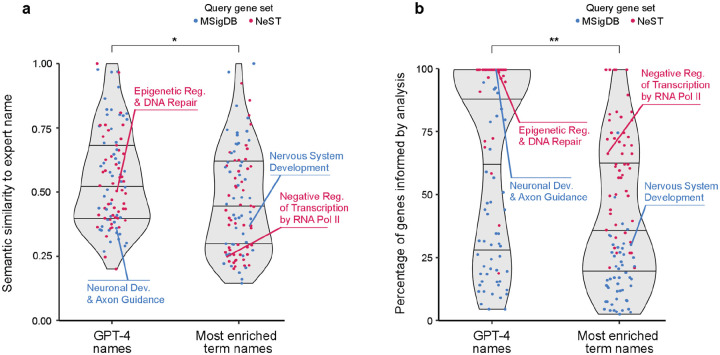
Evaluation of GPT-4 for analysis of gene sets discovered in ‘omics data. **a**, Violin plots showing the distribution of semantic similarity scores between GPT-4 names and expert-determined names (LEFT) or between most enriched GO:BP term name and expert-determined names (RIGHT) for gene sets discovered in ‘omics studies (points, n = 100). Blue: Gene sets based on expression clusters (MSigDB). Red: Gene sets based on protein interaction clusters (NeST). Horizontal lines denote median ± upper and lower quartiles. **b**, Distribution of the percentage of genes informed by GPT-4 analysis (LEFT) or gene set enrichment analysis (RIGHT). Plots elements same as panel a. For both panels, p-values are determined by Wilcoxon nonparametric two-sample comparison. * p=10^–3^; ** p=10^–7^.

For each gene set, we instructed GPT-4 to propose a name and a supporting analysis (Fig. 1b). For example, for the protein interaction cluster NeST:34 “Histone Modification,” GPT-4 proposed the name “Epigenetic Regulation and DNA Repair” along with eight paragraphs of supporting text (Table 3). We then compared the GPT-4 proposed name to the expert-determined name using semantic similarity (Fig. 1d, see Online Methods). Comparisons over the 100 ‘omics gene sets produced similarity scores in the range of approximately 0.2 to 1.0, with a median of 0.5 (Fig. 3a). To calibrate and assess these scores, we repeated the evaluation but replaced GPT-4 with a gene set enrichment analysis web service, Enrichr^8,34,35^, using the name of the most enriched GO term as the proposed name for the ‘omics gene set. In general, we observed that the similarities between GPT-4-proposed names and expert-determined names were significantly higher than the similarities between most enriched GO term names and expert-determined names (p = 1.2×10^−2^; Fig. 3a).

### Assessment and validation of GPT-4 supporting analysis text.

We next transitioned from analysis of GPT-4 naming to an examination of its supporting analysis text. We first noted that the fraction of genes mentioned in a supporting analysis spanned a very large range, from below 10% to 100% (‘omics gene sets; Fig. 3b). This fraction tended to be very high for the NeST protein interaction complexes in particular (median 91%). As a benchmark for comparison, we examined the fraction of gene set queries that were covered by the most enriched GO terms from the gene set enrichment analysis. Here, we found that this coverage was significantly lower than the fraction covered by GPT-4 analysis (p = 1.1 × 10^−7^; Fig. 3b). For example, GPT-4 presented arguments for the involvement of all 36 members of the gene× set “Hedgehog Signaling” (GPT-4 name: “Neuronal Development and Axon Guidance”) while the most enriched GO term, “Nervous System Development” covered 11 of the 36 genes (31%; Fig. 3 and Extended Data Table 2). Thus, GPT-4 actively assembles a broad synthesis of biological statements that typically include mention of the majority of genes in the set, without being limited to a predetermined gene set database.

In interpreting these results, an important concern with LLMs is their documented tendency to “hallucinate,” i.e., generate plausible but unverifiable or nonfactual statements^24^. To gain insight into the accuracy of the analysis text, we performed a structured review process for 50 paragraphs drawn from the analysis of 10 ‘omics gene sets (see Online Methods). For each paragraph, a human scientist attempted to verify each statement by a manual literature search. As a conservative criterion, we considered a paragraph “verified” if the reviewer found evidence for every statement. The reviewer also classified the paragraph as to whether, in their judgment, one or more of its statements supported the argument for the proposed name. Verification of accuracy and name support were evaluated independently, i.e., supportive paragraphs could include unverified statements. Of the 50 paragraphs evaluated, 39 were verified, and 44 were judged supportive (**Supplementary Table 3**). Examination of the 11 unverifiable paragraphs revealed a variety of misleading statements of 2 major types, related to (a) possible miscategorization of gene functions or (b) assertion of unverified biophysical interactions. In one case relevant to type (a), GPT-4 stated that “MMUT catalyzes the conversion of homocysteine to methionine” when in fact, it breaks down methionine to succinyl-CoA. Relevant to type (b), GTP-4’s statement “CDK5RAP1 also interacts with TRMT61B…” could not be verified, although such an interaction is plausible as both modify tRNA^36,37^. Thus, the verbose GPT-4 analysis paragraphs, with their many statements covering a high percentage of genes in a query set, appear to be largely but not entirely verifiable by human examination of the literature.

### Identification and assessment of relevant citations.

Given the need to verify statements in GPT-4’s analysis text, we developed a separate GPT-4-based system to identify publications that provide evidence for statements made in the analysis text (Extended Data Fig. 1, see Online Methods). This system was tested on the corpus of GPT-4 analysis paragraphs generated from the 100 ‘omics gene sets in Workflow 2. We recorded an average of 77 seconds to find and evaluate references for a gene set, approximately 9 seconds per paragraph (Extended Data Table 1). Out of 908 paragraphs, there were 187 for which the citation module did not detect any gene symbols or recognizable functions (e.g., see paragraphs 1 and 8 in Table 3). For nearly all remaining paragraphs (720/721), it returned at least one reference passing its evaluation criteria (see Online Methods).

To assess the relevance of these references, we performed a manual examination of selected citations (**Supplementary Table 4**) in which we required that the cited abstract must clearly and directly support one or more statements in the corresponding GPT-4 analysis paragraph. We did not require that the abstract be primarily about the statement; it was sufficient that a supporting fact was present. Of the 50 citations examined, we found that in 40 cases, the abstract and/or title indeed provided evidence for statements in the corresponding GPT-4 analysis paragraph. For example, the statements that GATA1 “regulates the expression of genes involved in RBC development, such as HBB, HBD, and ALAS2” and “is crucial for the differentiation of erythroid progenitor cells into mature RBCs” were supported by Zhang et al.^38^, “Intron 1 GATA site enhances ALAS2 expression indispensably during erythroid differentiation.”

## Discussion

The evaluations performed here suggest that GPT-4 indeed has notable potential as an automated assistant for understanding gene function. In the analysis of gene sets from the Gene Ontology (GO), GPT-4 produced highly similar names in 50% of instances, and in the majority of other cases, its name mapped to a term with a similar gene set, often a broader concept in the hierarchy. For gene sets emerging from transcriptomic and proteomic analyses, GPT-4 suggested names that compared favorably to gene set enrichment. Its accompanying analysis paragraphs provided supporting statements that were found to be largely factual, although its occasional generation of unverifiable statements (in approximately 22% of analysis paragraphs in our tests) suggests it should be coupled to some type of fact-checking or reference validation, whether automated or manual. In what follows, we discuss specific considerations related to computation time and scalability, comparison to gene set enrichment, interpretation of mismatches between GPT-4 and GO, and limitations of the present assessment.

### Relative to humans, GPT-4 is very fast in synthesizing common functions for gene sets.

The time for GPT-4 to name, analyze, and find references for a gene set was variable depending on the service’s response time but typically in the range of two to four minutes (Extended Data Table 1). By comparison, the time required for a human scientist to produce a comparable analysis is far greater, easily on the order of hours to build a sufficient understanding of the biological functions relevant to a gene set. Although this time estimate varies greatly depending on the analyst’s experience, the size of the gene set, and the specific gene functions involved, it includes (at minimum) multiple rounds of literature search, reading, and hypothesis generation, followed by authorship of a page of coherent summary analysis text. Interpretation of large numbers of gene sets, such as those produced by transcriptomics or proteomics (the technologies exemplified in Workflow 2), requires a significant commitment of human resources. In contrast, the detailed analysis of 100 gene sets performed in Workflow 2 was completed in roughly five hours.

### GPT-4 versus gene set enrichment: apples versus oranges.

While GPT-4 may be fast compared to human operators, it is slow compared to gene set enrichment, which is based on straightforward closed-form statistical equations requiring fractions of a second on a modern computer. The type of analysis offered by GPT-4 is very different from enrichment analysis, however. The goal of enrichment analysis is to identify the agreement of a gene set with those stored in reference databases. In contrast, GPT-4 predicts common functions for genes based on active reasoning over a large corpus of biomedical knowledge, producing results even in cases where the gene set does not resemble any recorded in reference databases. Accompanying the proposed functions is a rationale, given in the form of a summary analysis with arguments that can be critiqued, and subsequently validated or rejected by human investigators.

On the one hand, enrichment analysis could be said to be highly “transparent” because it uses well-defined statistical methods and documented reference databases of gene sets that researchers can review. In contrast, the knowledge accessed by the GPT-4 is not directly subject to definition or inspection, as it is embedded in the latent space of the model, and the mathematical calculation behind a given output is practically opaque. On the other hand, there is another sense in which an LLM analysis is extremely transparent because it presents a *de novo* narrative of facts and reasoning that considers all of the genes of interest, assisted by reference citation tools such as the one developed in this study. Ultimately, how an analysis was performed may be less relevant if the output is useful and its statements are supported by reasoned arguments and verifiable literature citations. Indeed, these are the criteria we apply to analyses produced by human researchers; we can see their output but not the operation of their minds.

### Mismatch of GPT-4 and GO names for gene sets: bug or feature?

For GO gene sets in which GPT-4 names were approximate matches to the names given by GO curators but conceptually more general (as many as one-fourth of queries we examined; Fig. 2c–e), it is possible this behavior should be called a GPT-4 failure mode. However, in detailed examinations of some of these instances, it remains unclear to us whether the GPT-4 name tends to be overly general or the GO name overly specific. In addition to the examples detailed above in our Results, consider the set of 36 genes annotated to GO:0048536: Spleen development, named “Regulation of cell cycle and apoptosis” by GPT-4 (**Supplementary Table 1**). We found that the majority of genes in this set, such as CDKN2B, BCL2L11, and ABL1, play established roles in cell cycle, apoptosis, and other processes with functions well outside of a specific organ system like the spleen^39–41^. In evaluating the choice of a broader rather than more specific name for such gene sets, we found the GPT-4 analysis paragraphs to be quite useful as a starting point for subsequent critical evaluation by human investigators. In comparison, it is not immediately clear how to access the rationale used by the GO curators in naming a GO term, although some of this information can be reconstructed indirectly by tracing the evidence codes and citations of each gene annotation.

### Limitations of this study.

It is important to stress that our evaluation of the GPT-4 model was based on single queries using prompts developed by informal experimentation. Further research should investigate more sophisticated prompting strategies such as “chain of thought”^42^ for gene set analyses and the use of methods that apply external tools and orchestrate multiple LLM interactions^43–49^, such as integrating literature searches into the LLM analysis rather than as a post hoc verification method. Our study also makes the implicit assumption that all gene sets emerging from a biological study have a coherent function to be summarized. What about gene sets that emerge erroneously from an ‘omics screen and are not functionally coherent, i.e., a type of false positive? One could imagine first asking the LLM to decide if the gene set has a common function and, even if the evidence is in favor, ask whether there are non-coherent genes that should be removed from the set or, alternatively, functionally similar genes that are missing and might be added. Finally, a critical consideration not yet addressed by our work is that gene functions are typically not universal but are specific to experimental conditions and biological contexts. Our study has not attempted to prompt LLMs with the contexts in which the gene set was discovered, information that might emerge as highly valuable to the quality of the analysis. Such knowledge is also absent from gene set functional enrichment tools, since their pre-existing mapping of gene sets to functions cannot encode the practically infinite space of biological conditions.

### Conclusions.

When applied to study gene function, one might have suspected that LLMs might produce statements, hypotheses, and references that hallucinate so uncontrollably as to be unusable. In fact, in our evaluations, GPT-4 typically did not, showing reasonable and often exemplary performance over a series of complementary benchmarks. We thus conclude that, given appropriate framing, the current general platform provides researchers with a new and powerful tool for gene set interpretation.

## Online Methods

### Choosing the large language model.

We used the ‘gpt-4–0314’ version of the OpenAI GPT-4 large language model for naming and analyzing gene sets, as a state-of-the-art model accessible using a well-defined API. GPT-4 has been reported to exhibit enhanced “steerability” over the earlier GPT–3 models^24^, meaning it can better infer a user’s intentions without extensive prompt tuning.

### Controlling the variability of GPT-4 responses.

The OpenAI API enables queries to set a “temperature” parameter that controls the variability of the generated response, with lower temperatures producing more reproducible and reliable responses^50,51^. Exploring the effect of temperature on GPT-4 analyses is outside the scope of this study, and therefore our queries used the lowest, most conservative/reproducible, temperature value = 0.0. In a manual inspection of repeated queries at temperature 0.0, we found that GPT-4 names and analyses were conceptually equivalent, but the specific text could vary, from near identity to considerable differences in phrasing. Additionally, we made our manual review process manageable by forcing the responses to be concise. For this purpose, we set the maximum number of tokens (roughly corresponding to words) in each response to be 1,000.

### Calculation of semantic similarity.

Semantic similarity between names was determined using the SapBERT model^52^. SapBERT produces embeddings of each name and then computes the cosine similarity between the embeddings, yielding a similarity score ranging from 0 (no similarity) to 1 (identical). SapBERT is a domain-specific language representation model that has been pre-trained on large-scale biomedical data, including Unified Medical Language System (UMLS), a massive collection of biomedical ontologies with 4M+ concepts. Since models like BERT are trained on vast amounts of textual data, they can learn general patterns and relationships and also capture context by considering surrounding words, providing a measure of similarity based on semantics rather than lexical matching. Although both SapBERT and GPT-4 are LLMs, they are two separate models with different purposes, model architecture, and training objectives and data. However, to address potential concerns that one language model was used in evaluating another, we performed the following test. For each ‘omics gene set in workflow 2, a panel of three humans conducted an independent and blinded evaluation of whether the GPT-4 determined name, or the most enriched term name, best matched the expert-determined name assigned in the MSigDB or NeST publications^12,32^. In 75/100 (75%) of these gene sets, the consensus of the independent panel matched the result of SapBERT semantic similarity testing (Extended Data Table 3). When SapBERT and the human panel disagreed, the disagreements were not significantly biased in favor of GPT-4 or gene set enrichment (p = 0.21, binomial test).

### Calibrating the similarity between GPT-4 names and GO names.

To evaluate the performance of the GPT-4 model in recapitulating GO names, we computed the semantic similarity between the GPT-4 name and the assigned name of the GO term query, using SapBERT as described above. We then performed this semantic similarity calculation for the same GPT-4 name against every other GO term name in the Biological Process branch (GO:BP), yielding a background distribution of semantic similarity scores. The actual and background similarities were then concatenated into a single list, sorted in descending order (largest to smallest), and the rank of the actual similarity was recorded and expressed as a percentile. This percentile score is thus the percentage of GO:BP term names that are less similar to the GPT-4 name than to the assigned name of the GO term query.

### Obtaining top enriched GO terms for the ‘omics gene sets.

We used the Enrichr web service to perform gene set enrichment. The most enriched GO:BP term for the queried gene set was obtained using the “gp.enrichr” function from GSEAPY package^53^ with the parameter settings {gene_sets = ‘GO_Biological_Process_2023’, organism=‘human’}. The best-matching GO term was selected based on the adjusted p-value and treated as the proposed GO name for the predominant biological process of the gene set.

### Identification and validation of relevant references (citation module).

The process of identifying and evaluating references for a given gene set involved several steps (Extended Data Fig. 1a–d). Firstly, we engineered a GPT-4 prompt to request two types of keywords from each analysis paragraph (as had been generated by an earlier GPT-4 query): (1) gene symbols explicitly mentioned in the paragraph and (2) up to three keywords associated with gene functions or biological processes, ordered by their importance. The prompt also gave the instruction that paragraphs should be skipped if they did not yield at least one gene symbol and one functional keyword and that the model should yield ‘unknown’ in those cases. The prompt to extract gene function keywords incorporated an example paragraph and corresponding answer as a guide. Following identification of gene symbols and function keywords, these were assembled in a series of logical query expressions, one per function, in which all gene symbols were combined using the ‘OR’ operator and linked with a function keyword by ‘AND’ (Extended Data Fig. 1b). The search then iterated over the query expressions, querying PubMed via its web API to find scientific publications in which the title or abstract matched the query expression, sorted by relevance (Extended Data Fig. 1c). The search process terminated when all query expressions were tried. Upon finding a match, the abstract of the relevant publication was extracted and provided to the GPT-4 model. This model was asked to assess whether the abstract agreed with one or more statements in the analysis paragraph (Extended Data Fig. 1d). A maximum of three references were returned per paragraph.

### Reviewer fact-checking of GPT-4 analysis text.

We performed a structured review of 50 paragraphs selected randomly from GPT-4 generated analysis text, gathered in a scoring table (**Supplementary Table 3**). In this analysis, a reviewer recorded scores for each paragraph based on two Boolean criteria, “Verified” and “Supporting,” in corresponding columns. Citations and other literature evidence were recorded in an “Evidence” column. The scoring rubric was as follows:

- Verified: Are all statements in the paragraph verified by literature review?
  - Verified paragraphs were scored as “1”, otherwise “0”.
  - Simple per-gene statements were checked against information from NCBI Gene content maintained by the National Library of Medicine, http://www.ncbi.nlm.nih.gov.
    - For example, “Oxytocin (OXT) is a neuropeptide hormone that binds to its receptor, oxytocin receptor (OXTR).” can be quickly verified by the NCBI Gene entries for the two genes.
    - If the NCBI entry verified one or more statements, the URL for the entry was added to the Evidence column, e.g., “NLM: OXT http://www.ncbi.nlm.nih.gov/gene/5020”
  - For statements not verified by NCBI Gene, the reviewer searched PubMed for publications to provide evidence for the statement.
  - Search strategies included:
    - Search using gene-keyword pairs, such as “TP53 cell cycle”.
    - For paragraphs that discuss multiple genes, search for review articles with phrases such as “acute phase response proteins.”
    - Search for family member proteins together, such as “TAS2Rs bitter taste”.
  - If no evidence for a statement could be found within roughly ten minutes of searching, this was noted in the Evidence column.
- Supportive: In the reviewer’s judgment, do any of the statements in the paragraph support the gene set name proposed by GPT-4?
  - Paragraphs judged supportive were scored as “1”, otherwise “0”.
  - Supportive paragraphs could include unverified statements.

## Extended Data

**Extended Data Table 1.** Estimated time and dollar usage for GPT-4 gene set analysis.

	Estimate time usage (second/gene set)	Estimate cost ($/gene set)
**Query gene set interpretation**	70	0.54
**Reference search and validation**	77	1.04

**Extended Data Table 2.** Representative names from ‘omics gene set analysis.

Expert determined name	GPT-4 determined name	Most enriched GO term name	# of Genes	% of Genes Captured	
				GPT-4	Most enriched GO term
Hedgehog Signaling	Neuronal Development and Axon Guidance	Nervous System Development	36	100%	31%
Notch Signaling	Notch Signaling Pathway	Notch Signaling Pathway	32	34%	100%
Ubiquitin regulation of p53 activity	Protein Degradation and Regulation	Protein Modification By Small Protein Conjugation	9	100%	67%
p53 regulation of cell cycle	Cell Cycle Regulation	Cellular Response To UV	5	100%	80%
Pancreas Beta Cells	Pancreatic Islet Cell Development and Function	Regulation Of Insulin Secretion	40	95%	23%
Interferon Alpha Response	Antiviral Immune Response	Defense Response To Virus	97	35%	34%
RAS-RAF-MAPK	MAPK/ERK Signaling Pathway	MAPK Cascade	7	100%	71%
ATM-independent DNA repair	DNA Damage Response and Repair	DNA Repair	32	97%	63%
Estrogen Response Early	Cell Proliferation and Differentiation	Vascular Transport	200	5%	5%
Nucleus	DNA Damage Response and Repair	Regulation Of Transcription By RNA Polymerase II	194	72%	51%

**Extended Data Table 3.** Evaluation of SapBERT for determination of semantic similarity.

SapBERT	Human panel	Number of gene sets
GPT-4* > Enrichment**	GPT-4 > Enrichment	47 (agreement)
GPT-4 > Enrichment	GPT-4 < Enrichment	15 (disagreement)
GPT-4 < Enrichment	GPT-4 > Enrichment	10 (disagreement)
GPT-4 < Enrichment	GPT-4 < Enrichment	28 (agreement)

* GPT-4 determined name

** Most enriched GO term name

## Figures and Tables

**Table 1.** Example of successful GO name recovery (GO:0006953 “Acute-Phase Response”).

**Genes** (n = 48)	A2M, AHSG, APOL2, APCS, ASS1, CD163, CEBPB, CNR1, CRP, EPO, EDNRB, F2, F8, FN1, HAMP, HP, IL1A, IL1B, IL6, IL6R, IL22, INS, ITIH4, LBP, MBL2, MRGPRX1, ORM1, ORM2, PLSCR1, PTGER3, PTGES, PTGS2, REG3A, REG3G, SAA1, SAA2, SAA4, SERPINA1, SERPINA3, SERPINF2, SIGIRR, TFRC, TFR2, TNF, TNFRSF11A, TNFSF11, TRPV1, UGT1A1
**GPT-4 Name** (Semantic Similarity)	Acute Phase Response (0.99)
**GPT-4 Analysis Paragraph**	
The most prominent biological process performed by this system of interacting proteins is the acute phase response (APR), which is a rapid inflammatory response to tissue injury, infection, or inflammation. The APR is characterized by the increased production of acute phase proteins (APPs) by the liver, which play a crucial role in the body’s defense against pathogens and tissue repair.	
1. Acute phase proteins: Several proteins in the list are well-known APPs, such as SERPINA1, SERPINA3, CRP, SAA1, SAA2, SAA4, ORM1, ORM2, HP, and A2M. These proteins are primarily synthesized in the liver and are involved in various aspects of the immune response, including opsonization, complement activation, and neutralization of proteases.	
2. Cytokines and inflammation: The list includes several cytokines and their receptors, such as IL1A, IL1B, IL6, IL6R, IL22, TNF, and TNFRSF11A, which are key mediators of inflammation and regulate the synthesis of APPs. For example, IL6 is a major inducer of the APR and stimulates the production of CRP and other APPs.	
3. Coagulation and fibrinolysis: Proteins like F2, F8, and SERPINF2 are involved in the coagulation cascade, which is activated during the APR to prevent blood loss and facilitate tissue repair. Additionally, the fibrinolytic system is regulated by proteins such as SERPINA1 and SERPINF2, which inhibit plasminogen activators and plasmin, respectively	
4. Iron homeostasis: Proteins like TFRC, TFR2, and HAMP are involved in iron homeostasis, which is altered during the APR to limit the availability of iron for pathogens. HAMP, also known as hepcidin, is an APP that regulates iron absorption and distribution by binding to ferroportin and inducing its internalization and degradation.	
5. Lipid metabolism and transport: Proteins like APOL2, APCS, and AHSG are involved in lipid metabolism and transport, which is essential for the immune response and tissue repair. For example, APOL2 is an apolipoprotein that plays a role in lipid transport and metabolism, while APCS is involved in the clearance of apoptotic cells.	
6. Cellular stress response: Proteins like CEBPB and HSP90AA1 are involved in the cellular stress response, which is activated during the APR to protect cells from damage and promote their survival. CEBPB is a transcription factor that regulates the expression of several APPs, while HSP90AA1 is a molecular chaperone that assists in protein folding and stabilization.	
In conclusion, the acute phase response is the most prominent biological process performed by this system of interacting proteins, as evidenced by the presence of numerous acute phase proteins, cytokines, and other proteins involved in inflammation, coagulation, iron homeostasis, lipid metabolism, and cellular stress response.	

**Table 2.** Best and worst GPT-4 names for GO terms by semantic similarity.

GO Name (# of genes)	GPT-4 Name (% of genes captured)	Semantic Similarity	Rank among all GO terms (n = 12,214)
**GPT-4 Names with Highest Similarity to GO Names**			
Mitochondrial tRNA Modification (9)	Mitochondrial tRNA Modification (100%)	1.00	1
Acute-Phase Response (48)	Acute Phase Response (56%)	0.99	1
Branched-Chain Amino Acid Catabolic Process (21)	Branched-Chain Amino Acid Catabolism (100%)	0.99	1
Homocysteine Metabolic Process (14)	Homocysteine Metabolism (93%)	0.99	1
Tryptophan Catabolic Process (9)	Tryptophan Catabolism (100%)	0.98	1
**GPT-4 Names with Lowest Similarity to GO Names**			
Ovulation From Ovarian Follicle (10)	Regulation of Cell Proliferation and Apoptosis (100%)	0.16	11,431
Positive Regulation of Prostaglandin Biosynthetic Process (7)	Inflammation Regulation (100%)	0.16	11,945
Positive Regulation of Protein Import Into Nucleus (39)	Hedgehog Signaling Pathway (97%)	0.15	11,324
Positive Regulation of Norepinephrine Secretion (4)	Oxytocin Signaling and Regulation (100%)	0.14	11,964
Positive Regulation of Histone H4 Acetylation (6)	DNA Repair and Transcription Regulation (100%)	0.13	11,887

**Table 3.** Representative analysis for protein interaction clusters (NeST:34 “Histone Modification”).

**Genes** (n = 30)	BARD1 BCL11B BRD4 CHD4 CREBBP DNMT1 DNMT3A DNMT3B E2F1 EMSY EZH2 HDAC1 HDAC2 HDAC3 HIST3H3 ING1 IRF2 KDM5A MTA1 PALB2 PARP1 PHF6 PRDM1 RB1 RUNX1 SMARCA1 TERT TP53 ZBTB2 ZNF217		
Analysis Method	Assigned Name	Semantic Similarity	# Genes Covered
**GPT-4**	Epigenetic Regulation and DNA Repair	0.50	30
**Gene set enrichment**	Negative Regulation Of Transcription By RNA Polymerase I	0.25	20
**GPT-4 Analysis Paragraph**			
The primary biological process performed by this system of interacting proteins is epigenetic regulation and DNA repair. The proteins involved in this system play crucial roles in modulating gene expression, chromatin remodeling, and maintaining genomic stability.			
1. Chromatin remodeling and histone modification: CHD4, SMARCA1, and ING1 are involved in chromatin remodeling, which is essential for regulating gene expression. HDAC1, HDAC2, and HDAC3 are histone deacetylases that remove acetyl groups from histones, leading to chromatin condensation and transcriptional repression. CREBBP is a histone acetyltransferase that adds acetyl groups to histones, promoting transcriptional activation. EZH2 is a histone methyltransferase that catalyzes the addition of methyl groups to histone H3, leading to gene silencing. KDM5A is a histone demethylase that removes methyl groups from histone H3, allowing for gene activation.			
Curtis et al., “The chromatin-remodeling enzymes BRG1 and CHD4 antagonistically regulate vascular Wnt signaling.” Molecular and cellular biology, 2012, pp. 1312–20.			
Larsen et al., “The chromatin-remodeling factor CHD4 coordinates signaling and repair after DNA damage.” The Journal of cell biology, 2010, pp. 731–40.			
Zhang et al., “Coordinated silencing of MYC-mediated miR-29 by HDAC3 and EZH2 as a therapeutic target of histone modification in aggressive B-Cell lymphomas.” Cancer cell, 2012, pp. 506–523.			
2. DNA methylation: DNMT1, DNMT3A, and DNMT3B are DNA methyltransferases that add methyl groups to cytosine residues in DNA, leading to gene silencing. This process is crucial for maintaining genomic stability and regulating gene expression.			
Dura et al., “DNMT3A-dependent DNA methylation is required for spermatogonial stem cells to commit to spermatogenesis.” Nature genetics, 2022, pp. 469–480.			
Zhang et al., “Structural basis for DNMT3A-mediated de novo DNA methylation.” Nature, 2018, pp. 387–391.			
Madsen et al., “An Important Role for DNMT3A-Mediated DNA Methylation in Cardiomyocyte Metabolism and Contractility.” Circulation, 2020, pp. 1562–1578.			
3. Transcriptional regulation: E2F1, IRF2, PRDM1, RB1, RUNX1, TP53, and ZBTB2 are transcription factors that bind to specific DNA sequences and regulate gene expression. BCL11B and ZNF217 are also involved in transcriptional regulation through their interactions with other transcription factors and chromatin modifiers.			
Choo et al., “The role of IRF1 and IRF2 transcription factors in leukaemogenesis.” Current gene therapy, 2006, pp. 543–50.			
Wang et al., “Prognostic analysis of E2F transcription factors E2F1 and E2F3 in four independent pediatric neuroblastoma cohorts.” BMC pediatrics, 2022, pp. 376.			
Owens et al., “Dynamic Runx1 chromatin boundaries affect gene expression in hematopoietic development.” Nature communications, 2022, pp. 773.			
4. DNA repair and genomic stability: BARD1, BRD4, EMSY, MTA1, PALB2, and PARP1 are involved in DNA repair pathways, such as homologous recombination and non-homologous end joining. These proteins help maintain genomic stability by repairing DNA damage and preventing mutations.			
Ray Chaudhuri et al., “The multifaceted roles of PARP1 in DNA repair and chromatin remodelling.” Nature reviews. Molecular cell biology, 2017, pp. 610–621.			
Tarsounas et al., “The antitumorigenic roles of BRCA1-BARD1 in DNA repair and replication.” Nature reviews. Molecular cell biology, 2020, pp. 284–299.			
Zada et al., “Parp1 promotes sleep, which enhances DNA repair in neurons.” Molecular cell, 2021, pp. 4979–4993.e7.			
5. Telomere maintenance: TERT is the catalytic subunit of telomerase, which is responsible for maintaining telomere length and preventing cellular senescence.			
Duan et al., “TERT-mediated and ATRX-mediated Telomere Maintenance and Neuroblastoma.” Journal of pediatric hematology/oncology, 2018, pp. 1–6.			
Wang et al., “The association of telomere maintenance and TERT expression with susceptibility to human papillomavirus infection in cervical epithelium.” Cellular and molecular life sciences : CMLS,2022, pp. 110.			
Goldstein et al., “Association of germline variants in telomere maintenance genes (POT1, TERF2IP, ACD, and TERT) with spitzoid morphology in familial melanoma: A multi-center case series.” JAAD international, 2023, pp. 43–51.			
6. Protein-protein interactions: PHF6 and HIST3H3 are involved in protein-protein interactions that modulate the activity of other proteins in this system.			
Wendorff et al., “Phf6 Loss Enhances HSC Self-Renewal Driving Tumor Initiation and Leukemia Stem Cell Activity in T-ALL.” Cancer discovery, 2019, pp. 436–451.			
Gan et al., “Chromatin-Binding Protein PHF6 Regulates Activity-Dependent Transcriptional Networks to Promote Hunger Response.” Cell reports, 2020, pp. 3717–3728.e6.			
Aillaud et al., “Tau Aggregation Inhibiting Peptides as Potential Therapeutics for Alzheimer Disease.” Cellular and molecular neurobiology, 2023, pp. 951–961.			
In summary, this system of interacting proteins plays a critical role in epigenetic regulation and DNA repair, ensuring proper gene expression and maintaining genomic stability.			

## Data Availability

Publicly available gene sets were used in this study. Gene Ontology (2022-10-07 release) is available at http://release.geneontology.org/2022-10-07/ontology/index.html. MSigDB can be found here: https://www.gsea-msigdb.org/gsea/msigdb and the selected NeST gene set is available to download from https://github.com/idekerlab/llm_evaluation_for_gene_set_interpretation/blob/main/data/NeST_table.txt.
